# Blood Lead Level in Children with Neurological Disorders

**Published:** 2018

**Authors:** Marzieh PARHOUDEH, Soroor INALOO, Mozhgan ZAHMATKESHAN, Zahra SERATISHIRAZI, Saeedeh HAGHBIN

**Affiliations:** 1 Department of Pediatrics, Shiraz University of Medical Sciences. Shiraz, Iran.; 2 neonatal research center, Shiraz, University of Medical Sciences. Shiraz, Iran.

**Keywords:** Lead poisoning, Brain diseases, Epilepsy, Cerebral palsy

## Abstract

**Objective:**

We aimed to investigate the blood lead level (BLL) in children with neurologic disorders of unknown causes and compare with normal children.

**Materials & Methods:**

In this prospective case-control study, 68 patients aged 1 to 18 yr with neurologic disorders of unknown causes, were referred to pediatric neurology clinics and wards, Shiraz, Iran selected during a 12 months period from Sep 2013. They were compared with 1:1 ratio, age, and sex-matched healthy children. BLL was checked from all participants using 3 cc heparinized venous blood sample. Level of ≥5 mcg/dl was considered toxic dose.

**Results:**

Totally, 136 children (68 cases and 68 controls) with mean ages of 5.20±4.12 and 4.18±3.86 yr, respectively, were enrolled. Mean BLL was higher in case group than in controls but the difference was not significant (*P*=0.84), though they were less than toxic levels in both. In addition, the difference in mean BLLs was not significant in terms of living place, sex, and age. Totally, 17.7% of the study sample had BLL ≥5 mcg/dl. The frequency of BLL ≥5 mcg/dl was significantly higher in case group (*P*=0.024) with an odds ratio 2.9 times higher (95% CI: 1.066-7.60).

**Conclusion:**

Strategies in public health must focus on practicing primary and secondary preventions of lead exposure in children.

## Introduction

“Lead toxicity has been one of the most significant preventable causes of neurologic morbidity from an environmental toxin” (1). While the immediate health effect of concern in children is typically neurological, it is important to remember that childhood lead poisoning can lead to health effects later in life. The neurologic symptoms of lead poisoning can range from acute encephalopathy to developmental delay. Chronic high level of lead poses the risk of intellectual impairment, poor educational attainment, behavior changes, insomnia, hyperactivity, hearing loss, upper extremity weakness, and lower lifetime achievement (2, 3). Patients may also suffer from brain edema, headache, and seizure. 

Lead poisoning is most commonly seen in children from lower socioeconomic background. As children absorb 50% of the lead they ingest, compared with the 10% absorbed in adults, children are more prone to lead poisoning (4). Moreover, they are more likely to be exposed to lead from crawling around the floors and hand-to-mouth activities (5). The growing child's brain has much greater sensitivity to lead than adult brain, so exposure to lead can have significant permanent effects on it. The center for disease control (CDC) have recently considered a blood lead level (BLL) of <5 mcg/dl as being acceptable due to the observed clinical manifestations of lower levels of lead (6). In 2004, 16% of all children in the world were estimated to have blood lead levels (BLLs) >10 mcg/dL (0.48 micromole/L) with 90% of children with elevated levels living in low-income communities (1). Mean blood lead concentration was higher in children with neurologic disorders and febrile convulsion, compared to that in normal counterparts (7-9). 

The aim of this study was to understand serum lead level in children with different neurologic disorders and compare them with healthy ones.

## Materials and Methods

In this prospective case-control study, patients aged 1 to 18 yr admitted to the Pediatric Neurology or referred to Neurology Clinic, Shiraz, Iran, with various neurologic disorders including seizure disorders, developmental delay, cerebral palsy, encephalopathy, attention deficit hyperactivity disorder (ADHD) and neuropathy with unknown causes, during Sep 2013 to Sep 2014, were selected. The control group was chosen randomly from the age and sex-matched healthy children with normal growth and development referred to the pediatric clinics for the routine checkup. Any participants with a positive result on metabolic or TORCH (*Toxoplasma*, rubella, cytomegalovirus, herpes simplex) screening were excluded from the study in both groups. BLL was checked for all participants using 3 cc venous blood sample in heparinized tube following centrifugation process and reported as atomic absorption spectrometry method. The level of >5 mcg/dl was considered the toxic dose. The required sample size was calculated using Epi-Info ver. 6.04. The calculation was based on the data from screening program in the USA, assuming a case-control ratio of 1:1 and odds ratio (OR) of 5, with an 80% power of detecting the difference at 5% level of significance. 

The study was approved by Research Ethics Committee of Shiraz University of Medical Sciences, and conducted according to the principles in the Helsinki Declaration of 1975, as revised in Brazil 2013. All participants and their parents or guardians provided their written informed consents and assents for participation. 

Statistical analysis was performed using SPSS software 15.0 (SPSS, Inc. Chicago, IL). Continuous variables were presented as mean ±SD and compared using independent samples *t*-test. Categorical variables presented in frequency and corresponding percentages and their relationships were assessed by Pearson correlation. Moreover, Odds ratio and confidence intervals (CI) have also been calculated using regression analysis. For ethical considerations, ethical approval was issued by the Research the Shiraz University of Medical Sciences (92-5015) and relevant authorities for this study.

## Results

Overall, 123 patients were selected. However, it was reduced to 68 based on the inclusion and exclusion criteria. They were compared to 68 healthy, age and sex-matched children with normal growth and development. 

The case group mostly consisted of those with seizure and developmental disorders; 40 (59%) and 12 (18%) cases, respectively. Their mean age was 5.20±4.12 yr (range: 1- 17 yr) and male to female ratio of 1. Characteristics of the study groups along with their BLL are shown in Table 1. Patients in the case group were older than control ones but the difference was not statistically significant (*P*=0.73).

Blood lead levels in the groups are shown in Table 2. Mean BLL was higher in case group than controls but the difference was not significant (*P*=0.84). The most important point was that the mean levels were less than toxic level in both groups. The respective lowest and highest BLL values in control group were 0 and 15 mcg/dl, while 0.1 and 9.5 mcg/dl in case group.

Mean BLL in both case and control groups according to variables are demonstrated in Table 3. The differences in BLLs were not statistically significant, in terms of living place, sex, and age. 

Totally 17.7% of the study sample (24/136) had BLL ≥5 mcg/dl. The prevalence of BLL ≥5 mcg/dl was significantly higher in group with neurological disorders, compared to that in controls (*P*=0.024) with odds ratio is 2.9 times higher (95% CI: 1.066-7.60) (Table 4).

## Discussion

Although this study revealed no statistically significant difference in mean BLL between patient and control groups, they were below toxic levels (Table 3). However, the risk of having BLL ≥ 5 mcg/dL was 2.9 times more in patients with underlying neurologic disorders, compared to normal counterparts (*P*=0.024) (Table 4).

**Table 1:** Demographics of the studied population

**Demographic variables**		**Case**	**Control **(%)	***P*** **-value**
Age (mean± SD), yr		5.20±4.12	4.18±3.86	0.73
Gender (N, %)	Male	33(48.6)	32(47.1)	0.81
	Female	35(51.4)	36(52.9)	0.68
Living area (N, %)	Urban	39(57.3)	34(50)	0.70
	Rural	29 (42.6)	34(50)	0.58

**Table 2:** Mean blood lead level in Case group with different neurologic disorders and control group

	**N0. of patients (%)**	**Mean+ SD, mcg/dl**
**Control group**	68 (100)	2.71±2.29
**Case group:**	68(100)	3.23±2.63
Seizure disorders	40 (59)	3.40+2.78
Developmental delay and CP†	12 (18)	2.60+2.90
Encephalopathy	4 (6)	3.07+2.95
ADHD‡	9 (13)	2.57+1.78
Neuropathy	3 (4)	3.10+0.5

† CP: Cerebral palsy,

‡ ADHD: Attention deficit hyperactive disorders

**Table 3:** Mean blood lead levels in case and control groups according to different variables

**Variable**	**Case (mcg/dl)**	**Control (mcg/dl)**	***P***
Rural	3.324±2.65	2.668±2.52	0.485
Urban	3.162±2.66	2.765±2.09	0.319
Male	3.033±2.27	3.097±2.89	0.922
Female	3.417±2.96	2.378±1.56	0.071
<2 yr old	3.00±2.76	2.47+2.65	0.506
2-5 yr old	3.035±2.64	2.69±1.95	0.445
5-10 yr old	3.68±2.48	2.73±1.37	0.203
10-18 yr old	2.48±2.99	3.83±2.92	0.381

**Table 4:** Comparison of blood lead level≥5 mcg/dl in case and control groups

	**Case No. (%)**	**Control No. (%)**	***P***
**BLL< 5 mcg/dl**	51(75)	61(89.1))	0.024
**BLL>= 5 mcg/dl**	17(25)	7(10.1))	
**Total **	68 (100)	68 (100)	

The prevalence of lead toxicity has decreased in the United States since 1970s because of preschool screening programs, increased public awareness, and the removal of lead from gasoline and paint products (1, 6). In many parts of the developing countries, lead continues to be used in gasoline, pigments (e.g., in paint, cosmetics, and crayons), pottery glaze, solder, cooking vessels, and even medications. The normal hand to mouth activity of children causes them to be highly prone to lead toxicity. In addition, they absorb more lead from gastrointestinal mucosa than adults (4). A broad spectrum of biomedical effects has been found to be associated with lead, but the most critical ones are those related to heme-biosynthesis, erythropoiesis, and nervous system function. Lead negatively affects children’s cognitive abilities and behavior, and defects have been observed at BLLs well below the action level of 5 mcg/dL set by the CDC (10, 11).

Different BLLs were associated with cognitive and developmental problems. Children with the BLL of 10-24.9 mcg/dl had a lower mental development index, compared with those with BLL 0-9.99 mcg/dl (12).

Children diagnosed with ADHD had a higher lead level, compared with the control group (7.2 vs.7.18 mcg/dl) the difference was not statistically significant though (13). The association of lower levels of lead (<5 mcg/dl) with the ADHD was also reported (14). A neuropsychology study in Cambridge comparing the preschool children being exposed to lead level <10 mcg/dl with their counterparts not being in contact with lead, the former group developed problems in doing their homework, maintaining attention and inability to restrain their automatic behaviors (15). A study examining children shortly after birth showed that apart from global intelligence, language-based functions were often found to be affected. Integrated postnatal rather than prenatal exposure is particularly deleterious, and no clear-cut effect threshold was found (16). However, in the present study, no statistically significant difference in BLL among case and control groups was detected. However, mean BLL≥5 mcg/dl was more prevalent in the group with neurologic disorders (*P*=0.024). As Shiraz is less industrialized with less air and water pollution, lower level of lead in children’s blood was observed. Moreover, use of lead-based paints has been banned in Shiraz since 10 yr ago.

The current study did not show any significant difference in mean BLL between two groups in terms of age, sex and place of living. The reason could be insufficient sample size to evaluate the concurrent effect of these three variables. A study on 230 rural and 272 urban in south US demonstrated that rural children had lower BLL, compared with urban children (17). In contrast, in a study in North Carolina, the prevalence of lead poisoning was significantly higher in rural areas (18). These differences could be explained by varying amount and use of lead among different nations and areas. In Iran, lead poisoning is more of a concern in urban areas due to the effect of industrialization. Likewise, the lead level is higher in Tehran, the capital city of Iran, compared with Shiraz, which is a less industrialized city (19).

In US during 1976 to 1980, children aged 24 to 48 months old with lead poisoning may not be identified due to lack of screening (20). Although preschool age was thought to be a risk factor for lead poisoning, children in this age group were also brought more to psychologists by their parents due to inattention problems and learning disabilities at school. Furthermore, the preschool children are more susceptible to toys related lead poisoning (20).

The present study demonstrated that the frequency of patients with BLL≥5 mcg/dl was significantly higher in groups with neurologic disorders than control one (*P*=0.024), consistent with some other studies (7-9). If on further inquiry these associations are found to be causal, lead exposure may represent a modifiable risk factor for this common condition of childhood. Lead poisoning is one of the most common and entirely preventable pediatric problems (1), strategies in public health must focus on practicing primary and secondary prevention of lead exposure in children. 

The case group in this study included patients with seizure disorders, developmental delay and cerebral palsy, ADHD, encephalopathy and neuropathy. Patients with cerebral palsy, seizure disorders, ADHD and acute encephalopathy of unknown origin had higher levels of BLL (7-9).

This study was indicative of the toxic effects of lead on the nervous system. Although a level of 5 or higher is considered toxic, no level of lead is normal or fine in children. Therefore, it is important to take measures to control the level of lead in the environment to the bare minimum in order to help minimize its side effects and prevent its poisoning.

## References

[1] World Health Organization (2010). Childhood lead poisoning.

[2] Eubig PA, Aguiar A, Schantz SL (2010). Lead and PCBs as risk factors for attention deficit/hyperactivity disorder. Environ Health Perspect.

[3] Davis DW, Chang F, Burns B, Robinson J, Dossett D (2004). Lead exposure and attention regulation in children living in poverty. Develop Med Child Neurol.

[4] Cohen SM (2001). Lead poisoning: a summary of treatment and prevention. Pediatr Nurs.

[5] Charney E, Kessler B, Farfel M, Jackson D (1983). Childhood lead poisoning: a controlled trial of the effect of dust-control measures on blood lead levels. N Engl J Med.

[6] CDC (2013). Morbidity and mortality weekly report (MMWR), blood Lead Levels in children aged 1–5 years — United States. MMWR Surveill Summ 1999–201.

[7] Kumar A, Dey P, Singla PN, Ambasht R, Upadhyay S (1998). Blood lead levels in children with neurological disorders. J Trop Pediatr.

[8] Lewendon G, Kinra S, Nelder R, Cronin T (2001). Should children with developmental and behavioral problems be routinely screened for lead. Arch Dis Child.

[9] Khosravi N, Izadi A, Noorbakhsh S (2014). Assessments of blood lead levels in children with febrile convulsion. Med J Islam Repub Iran.

[10] Guo P, Xu X, Huang B (2014). Blood lead levels and associated factors among children in Guiyu of China: a population-based study. PLoS One.

[11] Eubig PA, Aguiar A, Schantz SL (2010). Lead and PCBs as risk factors for attention deficit/hyperactivity disorder. Environ Health Perspect.

[12] Mendelsohn AL, Dreyer BP, Fierman AH (1998). Low level lead exposure and behavior in early childhood. Pediatrics.

[13] Daroogar S, Davari R, Kamran Lalbakhsh A (2012). The association of attention deficit hyperactivity disorder and blood lead level among children less than 10 yr old referred to Tehran hospitals between 2007 and 2010. Medical Sciences Journal of Islamic Azad University.

[14] Stiles KM, Bellinger DC (1993). Neuropsychological correlates of low-level lead exposure in school age children: a prospective study. Neurotoxicol Teratol.

[15] Koller K, Brown T, Spurgeon A, Levy L (2004). Recent developments in low-level lead exposure and intellectual impairment in children. Environ Health Perspect.

[16] Cohen CJ, Bowers GN, Lepow ML (1973). Epidemiology of lead poisoning A comparison between urban and rural children. JAMA.

[17] Norman EH, Bordley WC, Hertz-Picciotto I, Newton DA (1994). Rural-urban blood lead differences in North Carolina children. Pediatrics.

[18] Karrari P, Mehrpour O, Abdollahi M (2012). A systematic review on status of lead pollution and toxicity in Iran; Guidance for preventive measures. Daru.

[19] Brown MJ, DeGiacomo JM, Gallagher G (1990). Lead poisoning in children of different ages. N Engl J Med.

